# Factors associated with return to work of breast cancer survivors: a systematic review

**DOI:** 10.1186/1471-2458-14-S3-S8

**Published:** 2014-11-24

**Authors:** Tania Islam, Maznah Dahlui, Hazreen Abd Majid, Azmi Mohamed Nahar, Nur Aishah Mohd Taib, Tin Tin Su, MyBCC study group

**Affiliations:** 1 Centre for Population Health (CePH), Department of Social and Preventive Medicine, Faculty of Medicine, University of Malaya, Malaysia; 2 Department of Sports Medicine, Faculty of Medicine, University of Malaya, Malaysia; 3 Department of Surgery, Faculty of Medicine, University of Malaya, Malaysia

**Keywords:** Breast cancer, employment, work, barriers, facilitators, return to work (RTW)

## Abstract

**Background:**

The breast cancer survival rate is the highest among all types of cancers, and survivors returning to work after completing treatment is extremely important in regards to economy and rehabilitation. The aim of this systematic review study is to identify the prevalence of breast cancer survivors who return to work (RTW) and the factors associated to RTW.

**Methods:**

A computer based literature search was carried out. "PubMed, Cochrane Library, Embase, Web of Science, and Science Direct" databases were searched systematically. Our search strategy identified a total of 12,116 papers of which 26 studies met the inclusion criteria and quality assessment. These were original papers published between January 2003 and January 2013.

**Results:**

The trends in RTW differ among countries for the breast cancer survivors. The time to RTW after successful cancer treatment also varies among the countries and by ethnicity. The prevalence of the RTW varies from 43% to 93% within one year of diagnosis. The prevalence of the RTW for the Netherland is the lowest in the world (43%). The United States survivors showed the highest RTW (93%) within 12 months of the diagnosis. Numerous barriers and facilitators were identified as factors that affect RTW. For instance, socio-demographic factors such as education and ethnicity; treatment oriented factors such as chemotherapy; work related factors such as heavy physical work; disease related factors such as poor health condition and fatigue; and psychological factors such as depression and emotional distress, act as barriers of RTW. In contrast, social, family, employer support, and financial independency emerge as key facilitators in enabling breast cancer survivors to return and continue work.

**Conclusion:**

Minimising these identified barriers and strengthening these facilitators could further improve the work condition and increase the percentage of RTW among the breast cancer survivors.

## Background

Breast cancer is the most common cancer among women in high-, middle-, and low-income countries [1,2]. Studies have indicated that a majority of the women diagnosed belong to the working age group [3,4]. The survival rates on the other hand have improved significantly, especially in the high-income countries [5,6] because of advancement in cancer diagnosis and new cancer treatment regimes. Evidently, participation of female labour-force has increased over the last decades in high-, middle-, and low-income countries [2,7]. Hence, the increased survival rates warrant attention for breast cancer survivors to RTW [8,9]. For cancer survivors, RTW also underlines return to normal activities, social recovery and a positive step towards an improved quality of life, as well as rehabilitation after treatment.

Socio-demographic factors, patients psychological and disease condition, treatment related factors, support from employer, self-efficacy and rehabilitation influence cancer survivors' work ability [2,10-13]. The literatures related to RTW among breast cancer survivors used both qualitative [2,10,14-18] and quantitative research methods, such as cohort [3,8,9,19-26], longitudinal [6,27-29], cross-sectional [13,30,31] and randomised trial [32]. The qualitative studies emphasised on patients' psychological conditions, social as well as personal needs, and generated in-depth themes of the experience that survivors face in their workplace. In contrast, the quantitative studies stressed on the prevalence of employment, factors associated with RTW of cancer survivors across a large number of subjects. Hence, by combining both qualitative and quantitative methods, a thorough study could offer a unique means of examining these relationships.

There are two systematic reviews [11,12] on RTW among different types of cancer survivors that combined different types of cancers. To the best of our knowledge, only one systematic review paper [11] and one critical review paper [33] focused solely on breast cancer survivors. However, the systematic review paper focused on the effect and characteristics of four interventional studies on RTW among breast cancer survivors [11]. The critical review paper was focused on the factors associated with RTW among breast cancer survivors and the effectiveness of the conventional intervention strategies. The study focused on the North-American and European survivors from the perspective of disability management (DISM) [33]. To date, no systematic review has examined both qualitative and quantitative studies that emphasised on the prevalence and barriers to RTW in breast cancer survivors. Hence, the main objective of our systematic review is first, to assess the prevalence of RTW among breast cancer survivors and second, to identify facilitators and barriers associated with RTW.

## Methods

The PRISMA statement (Preferred Reporting Items for Systemic Reviews and Meta-Analyses) was followed as a formal guideline for this review.

### Search strategy

The criteria for the literature search for this review were the original papers published in English in peer-reviewed journals between January 2003 and January 2013. The search strategy involved the use of the following databases: PubMed, Cochrane Library, Embase, Web of Science, and Science Direct using extensive keywords search: "breast cancer", "return to work", "survivors", "employment", "employment status", "work" and "work activity".

### Selection of studies

Article titles that were identified during the initial search are first, screened by two independent reviewers--the primary reviewer (TI) and the second reviewer (TTS). Next, the selected titles are re-examined in the abstract review stage whereby two reviewers (as mentioned above) independently assess each abstract. In the third stage, the full text papers deemed as relevant based on the abstracts are obtained and further evaluated by two reviewers in terms of relevancy, quality and inclusion/exclusion criteria. A third reviewer (MD) is conferred and responsible to make the final decision should the initial reviewers disagree about an inclusion of a study. In addition, the reference lists of the selected articles are further reviewed to find other relevant studies, particularly those that were not identified in the initial search.

### Inclusion criteria

We included the articles that had following the properties: (i) were original articles using either quantitative, or qualitative methodology; (ii) reported prevalence of RTW or NRTW in quantitative studies (iii) focused on factors related in return to work among breast cancer survivors.

### Exclusion criteria

We excluded articles that (i) reported on all types of cancers and did not specify the breast cancer; (ii) were Protocol, Review article and Meta-Analysis; (iii) concentrated on work related breast cancer risk factors (e.g. industrial or environmental risk factors); and (iv) reported results based on the assessment of medical staff and employers or colleagues instead of breast cancer patients.

### Types of studies

Both qualitative and quantitative studies are included.

### Types of outcome measures

Studies that measured work related outcomes such as: (a) partial or full return to work, (b) absenteeism, (c) work disability, (d) employment status, (e) prevalence of return to work, among breast cancer survivors.

### Data extraction and quality assessment

Data were extracted from the included papers by one reviewer (TI) and checked for accurateness by the second reviewer (TTS). Disagreement in data extraction between reviewers was resolved by consensus. The extracted data included: first author, year and journal of publication, country, study design, sample, percentage of the population who RTW, factors associated with RTW or facilitator and barrier of RTW or employment and other comments on RTW. For our qualitative and quantitative studies we have used different quality assessment tools. National Critical Appraisal Skill Programme (CASP) Appraisal tool was used for qualitative studies [34]. For the quality assessment of cohort, cross-sectional, randomised trial, and longitudinal studies we consecutively used the New Castle Ottawa Scale (NOS) [35], British Sociological Association (BSA) Medical Sociology Group [36], Jadad scale [37], and Quality Assessment Tool for Systemic Reviews of Observational Studies (QATSO) [38]. More details about these quality assessments are described in Additional file 1.

## Results

**Figure 1:**
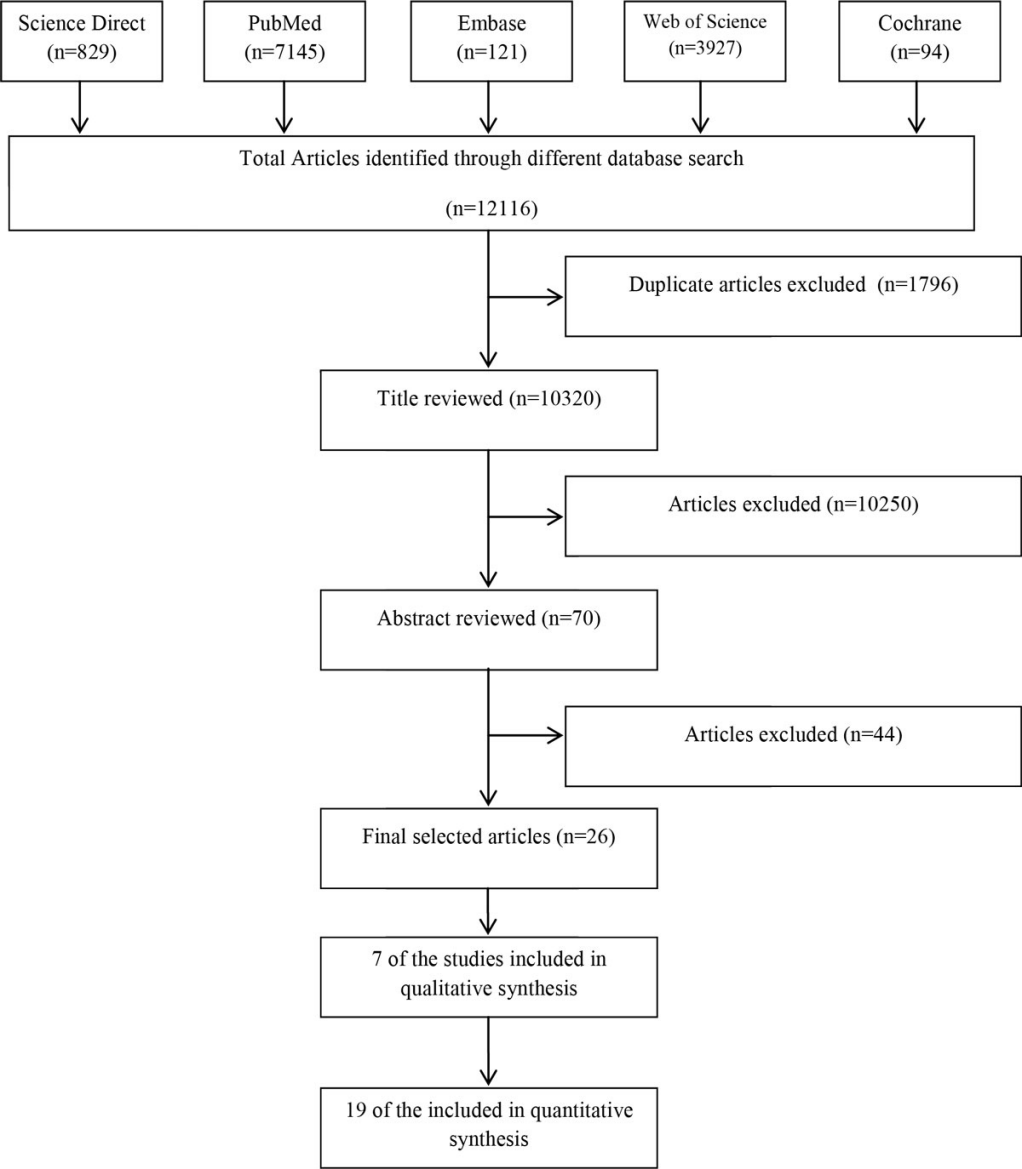
**Flow chart showing inclusion/exclusion of individual articles (or studies) for systematic review**.

Our search strategy identified a total of 12,116 articles; among them 1,796 articles were excluded due to duplication. 70 potential articles were identified based on the relevance of abstracts. Following a thorough review of the full text articles and after quality assessment, 26 articles were eligible for inclusion (Figure 1) [2,3,6,8-10,13-32]. Detailed findings of these articles are shown in Table 1 and 2.

**Table 1:** Summary characteristics (study design, facilitator & barrier of return to work ) of included studies.

Study	Country	Participants/ Sample	Study design	Facilitator for employment & RTW	Barrier of employment & RTW	Controversial/Doubtful/ Other comments on RTW
**A. Quantitative Study**						

E. Ahn et al, 2009, Breast cancer Res treat	South Korea	Breast cancer survivors N = 1594 & comparison group N = 415, female age 20 - 60 yrs	Cross sectional study (data from the five hospital-based breast cancer registries)	Age 40 - 49 No spouse, widow or divorced	Age <40 Low level of education Low household income Women lived with spouse *Fatigue & exhaustion Multiple co-morbidities Advance disease stage & more extensive surgery (e.g. mastectomy) Reduced work related abilities Decrease wages, reduced working hours,	House hold income may be both a cause or result of unemployment Socio-cultural factors as well as certain clinical characteristics influence the decisions of Korean women to return or not to RTW

C. Roelen et al, 2011, Breast Cancer Res Treat	Netherland	Breast cancer patients N = 492 (2008) Breast cancer patients N = 398 (2002), women age <40 - >50 yrs	Longitudinal study (ArboNed register data from 2002 to 2008)	Not described	Not described	The proportion of partial RTW was stable around 70% from 2002 to 2008 but full RTW was decreased from 52% in 2002 to 43% in 2008 -Change in Dutch disability policies in 2004 may be responsible for the decrease full RTW

S. Q. Fantoni et al, 2010, J Occup Rehabil	Northern France	Cancer survivors N = 379, age 18 - 60 yrs	Retrospective cohort study	Higher education women with no husband, moral support from friends and family moral support from the colleagues	Older age (>50 years), lower educational level, fatigue, pain chemo & radiotherapy, lymphoedema psychological constrains lack of moral support from the colleagues or employers	The self-perceived factors must be consider: first to help support these women during their sick leave and second to initiate a work resumption support process which takes into account both the person and her environment.

A. Johnsson et al, 2007, Acta Oncologica	Sweden	Pre-menopausal breast cancer patients N = 270, female age 29 - 54 yrs	Randomized trial	Not described	Advanced tumor stage adjuvant endocrine therapy Chemotherapy Not able to work same extent as previously.	Age, education level, marital status, under age children has no association with RTW

J. A. Hansen et al,2008, J Occup Environ Med	U.S.A	Breast cancer survivors N = 100, female mean age 49.5 years (range 20 - 70) & non-cancer comparison N = 103, mean age 39.8 years (range 20 - 70)	Cross sectional study	Not described	**Fatigue, work limitations depression	Demographic, medical status, and treatment variables were not related to work limitation

K Carlsen, 2013, Acta Oncologica	Denmark	No of cancer survivors = 170 & cancer free control N = 391, age 35 - 64	Cross sectional	Reduce work load, support from supervisors	low income fatigue reduced work ability Poor support from the colleagues and supervisors	Work ability of long-term breast cancer survivors who are disease-free and back in work is impaired in comparison with that of cancer-free women.

M. Drolet et al, 2005 CMAJ	Canada	Breast cancer survivors N = 646 & comparison group: N = 890, female age range 18 - 59 years	Retrospective cohort study	Financial burden Not belonging to a union self-employed, white-collar job	Patients who took chemotherapy, belonging to a union were more likely absented 4 weeks or more from their work	-
M. Drolet et al, 2005 Journal of clinical Oncology	Canada	Breast cancer survivors N = 646 & comparison group: N = 890, female age range 18 - 59 years	Retrospective cohort study	High income(<$ 50,000)	Older age low income (<$ 20,000 compared with ≥$ 50,000 ) union membership	√ Adjuvant therapy (chemo or radiotherapy) did not predict work cessation √ Slightly more survivors were not working 3 years after diagnosis compared with non-cancer women (21% and 15% respectively)

R. R. Bouknight et al, 2006, Journal of Clinical Oncology	USA	Cancer survivors enrolled: N = 443, completed study 12 months patients: N = 416, mean age 50.8 years completed study 18 months patients: N = 407, mean age 50.9 years	Prospective longitudinal study, assessment was done at 12 & 18 months of cancer diagnosis	Younger age good health, early tumor stage workplace accommodation	older age, black race, low health status advanced tumor stage heavy lifting work, perceived employer discrimination	Chemotherapy had no effect on return to work

A. Johnsson et al, 2011, Work	Sweden	Cancer survivors : N = 102, female age 35 - 63 years	Cohort study, assessment was done at 6 weeks, 6 & 10 months after surgery	Higher life satisfaction with life as a whole (satisfaction with vocational situation, somatic health and psychological health)	Low satisfaction with vocational situation irradiation to breast/ chest wall, and regional nodes Chemotherapy** axillary node dissection	Age, educational level, marital status, manual work were not associated with RTW

V.S. Blinder et al, 2012, Cancer	USA	Low income Latinas and Non-Latina white breast cancer survivors: N = 290, Latina survivors: N = 179, age 32-65 years & Non-Latina Whites survivors: N = 111, age 26-85 years	Longitudinal study, assessment was done at 6, 18, & 36 months of cancer diagnosis	More no of children at home to help with daily tasks, social support	**Low income Manual work, chemotherapy & higher comorbidity	Neither low-income Latinas nor non-Latina Whites approached the 80% rate of RTW seen in rich white cancer survivors

R. M. Villaverde et al, 2008 Occupational Medicine	Spain	Cancer survivors: N = 96, mean age 47 years (range 22 - 65 years)	Cohort study	Self-employed helpful co-workers and employers	Fatigue lymphodema, comorbidity, advanced stage disease	None reported job discrimination

E.Hedayati et al, 2012 Scand J Caring Sci	Sweden	Cancer survivors: N = 44, women age 40 - 64 years	Cohort study	Other adjuvant therapy except for chemotherapy	*chemotherapy advanced disease stage, lymph node involvement, positive Her2 status	Cognitive function do not predict RTW

B. Hauglann et al, 2012, J Cancer Surviv	Norway	Breast cancer case: N = 1548 and cancer free controls : N = 1548, age <50 -≥50 years	Cohort (National register based controlled cohort study)	Not described	Reduced income, reduce work ability early disability pension	At the end of the observation period, employment rates were higher in non-disabled patients than in non-disabled controls (82% vs.77%, *p *= 0.008)

E. Maunsell et al, 2004, journal of the National Cancer Institute	Canada	Breast cancer survivors: N = 646 comparison group: N = 890, age 18-59 years	Cohort study (population-based retrospective cohort study)	Not belonging to a union, No health insurance coverage among the labour force participants	Own decision to stop working new cancer events job is too difficult	√ Older age did not negetively affect the work situation √ discrimination at work was rare. √ After 3years, slightly more survivors (21%) than women in the comparison group (15%) were unemployed (RR adjusted = 1.29; 95% CI 1.05-1.59)

F. Balak et al, 2008, J occup Rehabil	Netherland	Patients with early stage breast cancer: N = 72, mean age 49.2 years (18-65 years)	cohort study	Patients who did not receive adjuvant therapy	Fatigue, chemotherapy & multimodal treatment	√ Age of women is not related to RTW √ The time taken to RTW after early stage breast cancer was principally determined by the type of treatment.

S. Lillehorn et al,2012, Scandinavian Journal of Caring Sciences	Sweden	Breast cancer survivors: N = 56, mean age 49 years (range 31-60 years)	longitudinal study, repeatedly interviewed over a period of 18-24 months	Willingness/ self-motivation, normalcy, missing work place	Physical sickness, chemotherapy, fatigue, exhaustion, discouraging work environment	Potential interactive relationships between biomedical and psychosocial circumstances affecting the return to work process.

M.J. Hassett et al, 2009, cancer	USA	Cancer survivors with employed health insurance: N = 3233, age 44-63 years	cohort(population of employed insured women) study	**health insurance	Chemotherapy	Radiation therapy did not influence employment

A. Johnsson et al, 2009, Acta Oncologica	Sweden	Survivors with early stage breast cancer: N = 102, age 35 - 63 years	Prospective Cohort (early stage breast cancer) study	Good self-rated health, Being born in Sweden, high satisfaction with life, low demand in work situation	High demand job chemotherapy axillary lymph node dissection	Age, educational level, living with underage children, marital status, manual work were not associated with RTW

**B. Qualitative Study**						

C. Tiedtke et al, 2012, BMC Public Health	Belgium	Breast cancer participants: N = 22, mean age 46 years (range 40 - 55 years)	Qualitative study	For financial independence, Self-motivation, Normalcy, Good social environment	Anxiety, frustration, Assuming employer will not eagerly welcome, employers negative attitude,	Four matters are considered prior to RTW: (i) women want to leave the sick role and wish to keep their job;(ii) they consider whether working is worth the effort; (iii) they reflect on their capability; and(iv) they have doubts about being accepted in the workplace after returning

F. L. Tan et al, 2012, Asian Pacific J Cancer Prev	Malaysia	Cancer survivors N = 40, female age 18 - 60 yrs	Qualitative study	Social Support (More for Malay) regards for financial independence (more among Chinese) support from the employers	Over protective family, tiredness, fatigue, pain depression, worrying, frustrations high physical job demand, fear of potential environment hazards	Health professionals and especially occupational therapist should be consulted to assist the increasing survivors by providing occupational rehabilitation to enhance RTW among employed survivors

V.S. Blinder et al, 2012, J Community Health	U.S.A	Cancer participants N = 23,among them African American = 3 African-Caribbean = 5 Chinese = 5 Filipina = 4 Latina = 3 Non-Latina white = 3; female age range 29 - 63 years	Qualitative study	Normalcy & Acceptance to maintain a normal environment at work, family history of breast cancer social support from friends, family and colleagues	Appearance and privacy lower support from the employers	Financial strain prevent African-American to take more time off from their work African-Caribbean get support from their friends & family Acceptance of cancer is common in Chinese Latina group has more fear of death, Non-Latina white has more family history of cancer which helps them to accept it.

C. Tiedtke et al, 2012, J Occup Rehabil	Belgium	Flemish stakeholders cancer survivors N = 26	Qualitative study	Improve legislation	Varying stakeholder perspective, Belgian legislation which emphasis the patients or disability role	Motivated stakeholders can positively affect RTW

A. Johnsson et al, 2010, Eur J Cancer Care	Sweden	Cancer survivors: N = 16, female age range 44 - 58 years	Qualitative study	Strong wish to stay in the labour market support from the workplace	Change in outlook, Poor social support, Psychological ill health, Diminish work capacity, unclear work roles,	Support from the workplace is of great importance for a successful RTW

S.J. Tamminga et al, 2012, Scand J work Environ Health	Netherland	Breast cancer survivors: N = 12, age 28 - 51 years	Qualitative study	Importance of work support from the supervisors social support	Temperment, feeling guilty treatment itself, having another co-morbidity slow or insufficient recovery over time physical workload, stressful job lack of support from colleagues, employers and/ or occupational physician	During initial RTW, physical & psychological side effect hampered work resumption. In the post RTW stage, work environment is the important factor.

M. Nilsson et al, 2011, Eur J Oncol Nurs	Sweden	Breast cancer survivors: N = 23, mean age 53 years (range 37 - 62 years)	Qualitative study	Support from the friends and family Support from the workplace, healthcare personnel flexibility in the implementation of sick rules	Lack of support from the colleagues and employer Disrespectful attitude of social insurance officers	* Three factors were identified which could be barrier or facilitators for RTW- i. The Adjustment to be arranged according to the survivor's requirements ii. Information (sickness insurance or side effects of treatment, benefit of being employment) sharing to the survivors iii. Attitudes (perceived and appraised) to the survivors by their surrounding people (Family, colleagues)

*RTW-return to work

**Table 2:** Prevalence of return to work among cancer survivors.

Author	Country	Study design	Participant and numbers of subjects	% of return to work (RTW)	% of return to work (RTW)						
					after treatment	6 months	12months	18 months	>18 months - 3yrs	No time limit / Others	Others comments on RTW

Ahn, 2009, Breast cancer Res treat	South Korea	Cross sectional study	Breast cancer survivors N = 1594 & comparison group N = 415, female age 20 - 60 yrs	after treatment 58.9% continued working	58.9%						

C. Roelen, 2010, Breast Cancer Res Treat	Netherland	Longitudinal study	Breast cancer patients N = 492 (2008) Breast cancer patients N = 398 (2002), women age <40 - >50 yrs	43% RTW within 1 yr (2008) of diagnosis			43%				52% RTW within 1 yr (2002) 43% RTW within 1 yr (2008)

S. Q. Fantoni, 2009, J Occup Rehabil	Northern France	Cohort study	cancer survivors N = 379, age 18 - 60 yrs	82.1% RTW after 18 months				82.10%			54.3%RTW in the 12 months after starting treatment

R. R. Bouknight, 2006, Journal of Clinical Oncology	USA	longitudinal study	Cancer survivors enrolled: N = 443, completed study 12 months patients: N = 416, mean age 50.8 years completed study 18 months patients: N = 407, mean age 50.9 years	82% & 83% RTW during 12, 18 months after diagnosis			82%	83%			At 12 months after breast cancer diagnosis, 18% and at 18 months 17% patients were not working

A. Johnsson, 2011, Work	Sweden	Cohort study	Cancer survivors : N = 102, female age 35 - 63 years	at 6 months 66% RTW & at 10 months 83% RTW		66%				83 % RTW after 10 months	

V.S. Blinder, 2012, Cancer	USA	longitudinal study	Low income Latinas and Non-Latina white breast cancer survivors: N = 290, Latina survivors: N = 179, age 32-65 years & Non-Latina Whites survivors: N = 111, age 26-85 years			27% Latina, 49% non-Latina (p = 0.0002)		Latina 45%, Non-Latina 59% (p = 0.02)	Latina 53%, Non-Latina 59% (p = 0.29)		<60% participant return to work within 3 yrs after diagnosis

R. M. Villaverde, 2008 Occupational Medicine	Spain	Cohort study	Cancer survivors: N = 96, mean age 47 years (range 22 - 65 years)	56% RTW at the end of treatment	56%						

E.Hedayati, 2012 Scandinavian Journal of Caring Science	Sweden	Cohort study	Cancer survivors: N = 44, women age 40 - 64 years	66% RTW 8 months after diagnosis &91 % RTW after 18 months				91%			

B. Hauglann, 2012, J Cancer Surviv	Norway	cohort	Breast cancer case: N = 1548 and cancer free controls : N = 1548, age <50 -≥50 years	At the end of observation period 82% nondisabled patients RTW (no time limit)						At the end of observation period 82% nondisabled breast cancer survivors RTW (no time limit)	At the end of observation period (9 yrs), employment rates were higher in non-disabled pts than non disabled controls(82% vs. 77%, p = 0.008)

E. Maunsell, 2004, journal of the National Cancer Institute	Canada	retrospective cohort study	Breast cancer survivors: N = 646 comparison group: N = 890, age 18-59 years	79% of cancer survivors working 3 yrs later						79% of cancer survivors working 3 yrs later	After 3 yrs more breast cancer survivors (21%) than women in comparison group (15%) were unemployed

S. Lillehorn,2012, Scandinavian Journal of Caring Sciences	Sweden	longitudinal study	Breast cancer survivors: N = 56, mean age 49 years (range 31-60 years)	29% after 6 months, 55% RTW after 12 months and at 18 months 57% RTW		29%	55%	57%			Including part time job 77% women RTW 12 months after diagnosis

M.J. Hassett, 2009, cancer	USA	cohort study	Cancer survivors with employed health insurance: N = 3233, age 44-63 years	93% women were still working 12 months later			93%				

A. Johnsson, 2009, Acta Oncologica	Sweden	cohort study	Breast cancer survivors: N = 102, age 35 - 63 years	59% women RTW 10 months after surgery						59% women RTW 10 months after surgery	

M. Drolet, 2005 CMAJ	Canada	retrospective cohort study	Breast cancer survivors N = 646 & comparison group: N = 890, female age range 18 - 59 years								85% of breast cancer survivors were absent 4 wks or more from work 1 yr after diagnosis

M. Drolet, 2005 Journal of clinical Oncology	Canada	retrospective cohort study	Breast cancer survivors N = 646 & comparison group: N = 890, female age range 18 - 59 years								21% of breast cancer survivors were not working 3 yrs after diagnosis

F. Balak, 2008, J occup Rehabil	Netherland	cohort study	Patients with early stage breast cancer: N = 72, mean age 49.2 years (18-65 years)								35% were absent longer than 1 yr and 4 patients did not returned to work within 2yrs after diagnosis

The background characteristics (study design, sample, factors associated with return to work related to facilitators or barriers of return to work, etc.) that are identified in these articles are shown in Table 1. Out of the 26 studies, seven are qualitative studies and 19 are quantitative studies. Among the quantitative studies; 11 are cohort, four longitudinal, three cross-sectional and one randomised trial study. Out of the 26 articles, 16 studies are from the Europe continent [3,6,8,10,13-15,17,18,21-24,26,29,32], eight studies are from the North America continent (USA and Canada) [9,16,19,20,25,27,28,31], and only two studies are from the Asia continent [2,30].

### Factors associated with return to work

#### Socio-demographic factors

Among the socio-demographic factors, survivor's age, education, marital status, number of children, ethnicity, household income and existence of relationship with surrounding people are important contributors that have relation to RTW. Younger age [28], higher education, marital status-single, high-income [14,19], positive social support from friends and family increase the likelihood of return to work [8,16,18,27]. Breast cancer survivors, who are single, divorced or widowed preferred to return to their work [8,30]. However, financial insecurity may also be the reason behind this.

Older [8,19,28] and married survivors [30] are less likely return to their work after the completion of their treatment. Marriage was a negative predictor of employment among Korean women as they were not the principal earners [30]. Lower educational level [8,30], low household income [13,19,27,30], overprotective family, poor social support [2,17] are considered as the barriers for return to work among cancer survivors. Education level had a relation with the type of job, for example, low educated patients were more likely to work as manual workers and, therefore, engaged in heavy lifting jobs and experienced fatigues [27]. Low household income may be both a cause or result of unemployment [30].

However Ahn *et al*. [30], described that the Korean women aged 40 years and above compared to those aged lesser than 40 years old more likely kept their respective jobs after treatment. Some of the selected articles have found age [9,21,24,26,32], marital status [21,26,32], educational level and underage children [21,26] as factors that have no relation to survivors who RTW. Besides, different ethnicities have different impacts on cancer survivors [2,16]. For example, Non-Latina White survivors have more family history of breast cancer and this background has helped them to accept the notion of being diagnosed with breast cancer compared to the Latina group for example, who is more afraid of death. Similarly, the African-Caribbean and Malay group ethnics receive better support from their friends and family, which then positively affects their decision-making. Acceptance of cancer is also common among the Chinese ethnicity.

#### Disease related factors

The disease related factors include stage of cancer, physical fitness level, exhaustion, fatigue, tiredness, pain and multiple co-morbidities, among others. Early stage breast cancer and good self-rated health are important facilitators for RTW [26,28]. Fatigue and exhaustion were the most frequent problems reported by cancer survivors [2,6,13,24,30,31]. As such, fatigue is one of the most important factors that prevent cancer survivors to rejoin the workforce or reduce their capability to work. Furthermore, fatigue is also seen to affect patients psychologically. Advanced disease stage, new cancer episodes, tiredness, pain, other associated co-morbidities are other prevalent barriers of re-employment [2,8,14,30,32].

#### Treatment related factors

Treatment related factors refer to methods of treatment which survivors had gone through such as surgery and adjuvant therapies like chemotherapy, radiotherapy and hormonal therapy. A few studies have found that patients who received other adjuvant therapies except chemotherapy [23] or patients who did not receive any adjuvant therapy [24], return to work earlier.

Chemotherapy was found to be a major barrier in most of the quantitative studies [6,8,20,21,23-27,32] due to its side effects like nausea and vomiting [39]. Even after completing treatment, several side effects of chemotherapy such as depression, fatigue and cognitive dysfunction were said to persist and contribute to the work inability [21]. In addition to chemotherapy, extensive surgery such as mastectomy and axillary node dissection, irradiation to breast or chest wall, and regional nodes, hormone and radiotherapy were also found to be important barriers to RTW [8,21,26,30] as both surgery and radiotherapy to the regional nodes cause arm morbidity. And as a result, arm morbidity after breast cancer treatment reduces work capability and also causes psychological problem [32,40].

However, only few studies have found that chemotherapy and radiotherapy do not have an effect on RTW [19,25,28].

#### Psychological factors

There are several psychological factors which influence patients' decision making on RTW. Higher life satisfaction as a whole (satisfaction with vocational situation, somatic health and psychological health), willingness or self-motivation, normalcy and acceptance to maintain a normal environment at work, are some of such important factors.

Changes in emotional states such as depression, worry, frustration, fear of potential environmental hazards, and feeling guilty are potential barriers that influence survivors' decision making to RTW [2,8,14-17,31]. Physical appearance, privacy, poor social support in the workplace, and job discrimination, are factors associated with survivor's psychological ill health [2,17].

#### Work related factors

There are several work related factors, such as type of jobs (e.g. manual vs. desk job, stressful vs. non-stressful job), job facility, flexibility, support from colleagues and employers as well as perception of job importance by breast cancer survivors that motivate them to RTW. Almost all the articles have identified that positive and active support from employers and colleagues are the key facilitators to RTW [2,8,13,14,16-18,20,22,26,28]. Survivors who returned to work after treatment look forward to a flexible working schedule, less manual job and job security while they too have equal rights of a healthy worker. Clearly, these supports can be provided in different ways. For example, employers made changes to work schedules, thus making it easier for women to have their cancer treatment and additional arrangements to ease the workload [17,18]. Health insurance coverage provided by employers also plays an important role in RTW. According to Hasset *et al*. [25], 93% of insured women return to their work within 12 months after diagnosis in the USA. Interestingly, the USA does not provide free healthcare for its citizen unlike the UK and Canada. As such, most people in the USA rely on private healthcare institutions, thus, the fear of losing health insurance can significantly influence RTW after cancer treatment [30]. It is also found that, among the Canadian breast cancer survivors, women who belong to an employee union are more likely to be absent for some period than women who do not belong to a union [9,20].

Manual work, stressful job, lack of support from colleagues, employers and/or occupational physician, reduced working hours, decreased wages were factors identified that discourage survivors to re-enter their jobs [2,6,8,9,13-16,18,26-28,30]. Among these factors high job demand and negative or lack of support from the colleagues and employer emerge as primary barriers for survivors. On the other hand, national health insurance system [30] and early or longer disability pension [3,29] were found to delay or reduce RTW. One such example includes the Dutch disability policy that was amended in 2004, a distinct decline was noticed in RTW among breast cancer survivors [29] as a result of granted absence compensation for a period of two years instead of one year for sick people.

### Prevalence of return to work

Table 2 describes the detailed information of prevalence of RTW among breast cancer survivors. Out of the 19 quantitative papers, 13 papers revealed information on percentage of survivors who RTW and 3 papers showed the percentage who were absent from their work.

Different studies have elaborated different timing and percentage of survivors who return to their work. The prevalence of the RTW varies from 43% to 93% within one year of diagnosis. The prevalence of the RTW for Netherland is the lowest in the world (i.e.; after diagnosis 43% RTW). The USA survivors showed the highest RTW (93%) within twelve months of the diagnosis. There is wide range of time of RTW among cancer survivors, from six months to no specific time limit. The prevalence of the RTW for the low-income Latina in the USA was the lowest with 27% returning to work at six months [27]. Even after three years of diagnosis, only 53% of low-income Latina and 59% of Non-Latina White survivors return to their work. Another study within the USA have found that survivors with health insurance coverage showed the highest RTW (93%) 12 months after diagnosis [25]. Ethnicity, cancer stage during diagnosis, treatment, job type, income, insurance and quality of life were important drivers for this discrepancy in return to work [16,25]. Income related disparities in regards to quality of life could also help explain the difference in employment outcomes between low-, middle- or high-income survivors. Employment covered health insurance system also play an important role, as fear of losing health insurance significantly influences the decision to resume employment after cancer treatment [25].

## Discussion

The objective of this literature review is to provide an overview on breast cancer survivorship issues namely those related to employment and work. After an extensive literature review, we have identified 26 articles that have analysed the frequency of employment and return to work, absenteeism from work, factors related to employment, i.e. facilitators and barriers of returning to work. The prevalence of RTW varies from 43% to 93% within one year of diagnosis. Without a time limitation of one year, prevalence of RTW was low at 27% (low-income Latina ethnicity in the USA) to as high as 93% (among the USA cancer survivors with employment related health insurance). From this literature review, we have identified that white collar job, early tumour stage, self-motivation, normalcy and acceptance to maintain a normal life, support from the friends, family and workplace, employment related health insurance are the important factors that facilitate survivors' RTW. Conversely, low-income, on-going chemotherapy, fatigue and exhaustion psychological constrain, high job demand, poor support from the colleagues and employers are the potential barriers influencing cancer survivors not to resume their job.

Despite these challenges and barriers in RTW, our research noticed that a majority of cancer survivors do tend to return to their work in the long-run [6,21,25,28]. The notion of returning to the labour market is a sign of regained well-being and a reconnection to ordinary life. In fact, it is also seen as a positive step towards rehabilitation. However, it should be more cautious in some instances because survivors returning to work may be a reflection of lack of support, financial burden or fear of medical insecurity.

Cancer survivors return early if they have financial need for their treatment or family commitments. Health insurance system and benefits such as sick leave and disability pensions have great influence on survivors' RTW in both positive and negative way [2,9,25,29,30]. Each country may face unique challenges in RTW since breast cancer survivors from different countries have different psychology and socio-cultural make-up. For example, a study in the USA revealed, Chinese women were found to continue working throughout the treatment period [16]. This was attributed to better acceptance of cancer diagnosis amongst the Chinese group compared to the other ethnic groups such as the African, Filipino or Latina.

In the Asian countries, women tend to participate less actively in the workplace, become more committed to their families and are economically dependent on their significant others after marriage. A study in Malaysia [2] has found that, financial independence was mostly expressed among the Chinese ethnicity compared to Malay and Indian ethnics. Future studies should observe if the rate of RTW amongst Chinese women is higher compared to other races. Breast cancer survivors also tend to be younger in Asia than survivors in Western countries [30]. Thus the rate and factors associated with RTW may differ in Asian countries as to Western countries. Thus, we also suggest that additional studies should be done among different ethnic breast cancer survivors in Asia and Asian immigrants in Western countries.

### Strength

Our systemic review paper focused only on breast cancer and we have included both qualitative and quantitative papers. Qualitative articles focused on survivor's deep thoughts, psychological condition, feelings regarding work and these findings aided as we explored the perception of facilitator and barriers to RTW. Conversely quantitative studies, provided information on the prevalence of RTW and the factors that either supported or discouraged RTW, which increased the understanding and support towards a better environment of workplace for the breast cancer survivors. Most of the studies were moderate or high quality paper except for one paper. However, the paper was assessed by Jadad scoring and it has only two categories such as high or poor quality. There is no scoring for moderate quality paper.

### Limitation

Although we systematically searched the literatures, the papers that met with inclusion criteria and quality assessment were from high-income countries except for one study from low- and middle-income countries. Hence, factors that affect RTW among the breast cancer survivors in low- and middle-income countries remain scarce. This calls for more research on the issue of RTW and its socio-economic impact to prepare for the increasing cancer burden in Asia and low and middle resource countries. There is also lack of studies from other continents such as Africa, Australia and South America and this again is a drawback to understand RTW issue globally. We also could not conduct meta-analysis as different quantitative studies defined the RTW in different ways especially for timing. Besides, the categorizations for employment were inconsistent among the papers, for example, full-time or part-time or a combination of the both.

## Conclusion

Breast cancer survivors are more likely to continue working after the completion of their cancer treatment. Future research is needed to examine the prevalence of employment and factors that affect RTW among breast cancer survivors in low- and middle-income countries where these factors may be diverse and different. Increasing cancer burden in Asia warrants special attention as socio-cultural values, participation of female labour force, health insurance system, employment and law/environment varies to those of the Western countries. Besides, papers related to RTW should explicitly mention the "time" of RTW specifically as well as distinguish women who have a choice about RTW, and those who need to RTW out of any other option. We also believe that future meta-analysis, and studies from all over the world can further improve the understanding on the impact on RTW for the breast cancer survivors. Better knowledge on work related problem, cancer, and its treatments that induce physical, cognitive, psychological effect on survivors will help the related personnel to develop necessary interventions, and rehabilitation of the survivors. Consecutively, this effort can motivate and enhance the rate of RTW to a great extent.

## Competing interests

The authors declare that they have no competing interests.

## Authors' contributions

TI conducted the literature search. TI, TTS and MD were involved in both the selection of articles and data extraction. All authors contributed in design of the study and write-up of the manuscript.

MyBCC study group: Taib NA, Hussain SH, Dahlui M, Su TT, Bhoo-Pathy N, Ng CG, Majid HA, Nahar AM from University of Malaya and Murray LJ and Cantwell M from Queens University Belfast.

## Supplementary Material

Additional file 1 Summary of the Quality Assessment Tools Click here for file
