# Supplementary material for: Factors associated with return to work of breast cancer survivors: a systematic review
Source: BMC Public Health. 2014 Nov 24;14(Suppl 3):S8. doi: 10.1186/1471-2458-14-S3-S8 (PMC4251139; doi:10.1186/1471-2458-14-S3-S8)
Supplement: Additional file 1 — Summary of the Quality Assessment Tools [file 1471-2458-14-S3-S8-S1.pdf]

Additional file 1

Summary of the Quality Assessment Tools

Study Design	***Quality Assessment Tool***	***Quality Rating of study***
Cross Sectional	BSA Medical Sociology Group	Score 1-7<br>1-2 (Low)<br>3-5 (Moderate)<br>6-7 (High)
Randomised Trial	Jadad Scale *(Jadad et al 96)*	< 3 (Poor)<br>≥3 (High)
Cohort Study	Newcastle Ottawa Scale, NOS	Selection (Max 4 *)<br>Comparability (Max 2*)<br>Outcome / Exposure (Max 3*)
Longitudinal Study	The Quality Assessment Tool for Systematic Reviews of Observational Studies (QATSO)	External validity (1 item)<br>Reporting (2 items)<br>Bias (1 item)<br>Confounding (1 item)
Qualitative Study	National CASP Appraisal Tool	Score 1-10<br>1-3 (Low)<br>4-7 (Moderate)<br>8-10 (High)

Table: Summary of quality of included studies.

Author	***Year and Journal of Publication***	***Study Design***	***Quality Assessment Tool***	***Quality Rating of study***	***Comments***
E. Ahn *et al*	2009, Breast Cancer Res Treat	Cross Sectional	BSA Medical Sociology Group	7	High
K. Carlsen *et al*	2013, Acta Oncologica	Cross Sectional	BSA Medical Sociology Group	7	High
J.A. Hansen *et al*	2008, Occup Environ Med	Cross Sectional	BSA Medical Sociology Group	6	High
S.Q. Fantoni *et al*	2010, J Occup Rehabil	Cohort study	Newcastle Ottawa Scale, NOS	Selection ** Comparability * Outcome / Exposure **	
M. Drolet *et al*	2005, CMAJ	Cohort study	Newcastle Ottawa Scale, NOS	Selection **** Comparability * Outcome / Exposure ***	
M. Drolet *et al*	2005, Journal of Clinical Oncology	Cohort study	Newcastle Ottawa Scale, NOS	Selection **** Comparability ** Outcome / Exposure ***	
A. Johnsson *et al*	2011, Work	Cohort study	Newcastle Ottawa Scale, NOS	Selection *** Comparability * Outcome / Exposure **	
R.M. Villaverde *et al*	2008, Occupational Medicine	Cohort study	Newcastle Ottawa Scale, NOS	Selection * Comparability * Outcome / Exposure *	

E. Hedayati *et al*	2012, Scand J Caring Sci	Cohort study	Newcastle Ottawa Scale, NOS	Selection *** Comparability ** Outcome / Exposure ***	
B. Hauglann *et al*	2012, J Cancer Surviv	Cohort study	Newcastle Ottawa Scale, NOS	Selection **** Comparability ** Outcome / Exposure ***	
E. Maunsell *et al*	2004, Journal of National Cancer Institute	Cohort study	Newcastle Ottawa Scale, NOS	Selection **** Comparability ** Outcome / Exposure **	
F. Balak *et al*	2008, J Occup Rehabil	Cohort study	Newcastle Ottawa Scale, NOS	Selection *** Comparability * Outcome / Exposure ***	
M. J. Hasset *et al*	2009, Cancer	Cohort study	Newcastle Ottawa Scale, NOS	Selection *** Comparability * Outcome / Exposure ***	
A.Johnsson *et al*	2009, Acta Oncologica	Cohort study	Newcastle Ottawa Scale, NOS	Selection ** Comparability ** Outcome / Exposure **	
C. Roelen *et al*	2011, Breast Cancer Res Treat	Longitudinal study	The Quality Assessment Tool for Systematic Reviews of Observational Studies (QATSO)	External validity 1 Reporting 2 Bias 1 Confounding 0	Good
R. R. Brouknight *et al*	2006, Journal of Clinical Oncology	Longitudinal study	The Quality Assessment Tool for Systematic Reviews of Observational Studies (QATSO)	External validity 0 Reporting 1 Bias 1 Confounding 0	Fair
V.S. Blinder *et al*	2012, Cancer	Longitudinal study	The Quality Assessment Tool for Systematic Reviews of	External validity 1	Good

			Observational Studies (QATSO)	Reporting 2<br>Bias 1<br>Confounding 0	
S. Liillehorn *et al*	2012, Scand J of Caring Sci	Longitudinal study	The Quality Assessment Tool for Systematic Reviews of Observational Studies (QATSO)	External validity 1<br>Reporting 2<br>Bias 0<br>Confounding 0	Fair
A. Johnsson *et al*	2007, Acta Oncologica	Randomized Trial	Jadad Scale	2	Poor
C.Tiedtke *et al*	2012, BMC Public Health	Qualitative Studies	National CASP Appraisal Tool	9	High
F.L.Tan *et al*	2012, Asia pacific J.Cancer Prev	Qualitative Studies	National CASP Appraisal Tool	8	High
V.S Blinder *et al*	2012, Journal of Community Health	Qualitative Studies	National CASP Appraisal Tool	9	High
C. Tiedtke *et al*	2012, J Occup Rehabil	Qualitative Studies	National CASP Appraisal Tool	8	High
A. Johnsson *et al*	2010, Eur J Cancer Care	Qualitative Studies	National CASP Appraisal Tool	8	High
S.J Tamminga *et al*	2012, Scand J Work Environ Health	Qualitative Studies	National CASP Appraisal Tool	8	High
M. Nilsson *et al*	2011, European Journal of Oncology Nursing	Qualitative Studies	National CASP Appraisal Tool	8	High

QUALITY ASSESSMENT OF CROSS SECTIONAL STUDIES (BSA Medical Sociology Group)

Seven Quality Indicators:

1) **Appropriate Research Design**
2) **Appropriate Recruitment Strategy**
3) **Response Rate Reported**
4) **Sample Representative of Similar Population**
5) **Objective and Reliable Measures Used**
6) **Power Calculation/Justification of Numbers Reported**
7) **Appropriate Statistical Analysis**

**** Y=Yes N=No [Quality Indicators Met out of 7: 1-2 (Low)---- 3-5 (Moderate)----6-7 (High)]**

Author and year	Appropriate Research Design?<br><br>(Y/N)	Appropriate Recruitment Strategy?<br><br>(Y/N)	Response Rate?<br><br>(Y/N) %	Is Sample Representative? (All similar populations)<br>(Y/N)	Objective and Reliable Measures?<br>(Y/N)	Power Calculation/ Justification of Numbers?<br>(Y/N)	Appropriate Statistical Analysis?<br><br>(Y/N)	Quality Indicators Met<br><br>(out of 7)

QUALITY ASSESSMENT FOR QUALITATIVE STUDIES

Critical Appraisal Skill Programme (CASP):

Ten quality indicators:

1. Was there a clear statement of the aims of the research?
2. Is a qualitative methodology appropriate?
3. Was the research design appropriate to address the aims of the research?

4. Was the recruitment strategy appropriate to the aims of the research?
5. Were the data collected in a way that addressed the research issue?
6. Has the relationship between researcher and participants been adequately considered?
7. Have ethical issues been taken into consideration?
8. Was the data analysis sufficiently rigorous?
9. Is there a clear statement of findings?
10. How valuable is the research?

NEWCASTLE - OTTAWA QUALITY ASSESSMENT SCALE (NOS) OF COHORT STUDIES

Selection (Max 4 Stars)

1) Representativeness of the exposed cohort

a) truly representative of the average ________________ (describe) in the community *****

b) somewhat representative of the average _______________ in the community *****

c) selected group of users, e.g. nurses, volunteers

d) no description of the derivation of the cohort

2) Selection of the non exposed cohort

a) drawn from the same community as the exposed cohort *****

b) drawn from a different source

c) no description of the derivation of the non exposed cohort

3) <u>Ascertainment of exposure</u>

a) secure record (e.g. surgical records) *****

b) structured interview *****

c) written self report

d) no description

4) <u>Demonstration that outcome of interest was not present at start of study</u>

a) yes *****

b) no

Comparability (Max 2 Stars)

1) <u>Comparability of cohorts on the basis of the design or analysis</u>

a) study controls for ______________ (select the most important factor) *****

b) study controls for any additional factor ***** (This criteria could be modified to indicate specific control for a second important factor.)

Outcome (Max 3 Stars)

1) <u>Assessment of outcome</u>

a) independent blind assessment *****

b) record linkage *****

c) self report

d) no description

2) <u>Was follow-up long enough for outcomes to occur</u>

a) yes (select an adequate follow up period for outcome of interest) *****

b) no

3) <u>Adequacy of follow up of cohorts</u>

a) complete follow up - all subjects accounted for *****

b) subjects lost to follow up unlikely to introduce bias - small number lost - > ____ % (select an adequate %) follow up, or description provided of those lost) *****

c) follow up rate < ____% (select an adequate %) and no description of those lost

d) no statement

Assessing the Quality of Randomised Trial

JADAD ASSESSMENT TOOL

**JADAD SCORING CRITERIA**	Potential Score	Score Awarded
1_a. Was the study described as randomized?	+ 1	
$1_{b.}$ Was the method of randomization described and appropriate to conceal allocation?	+1	
$1_{c.}$ If described and inappropriate, describe:	-1	
$2_{a.}$ Was the study described as double blinded?	+1	
$2_{b.}$ Was the method of double-blinding described and appropriate to maintain a double blinding?	+1	
$2_{c.}$ Was the method of blinding inappropriate?	-1	
3. Was there a description of withdrawals and drop outs?	+1	
**Final Score (0-5)**	5	_ /5

QUALITY ASSESSMENT OF LONGITUDINAL STUDIES

The Quality Assessment Tool for Systematic Reviews of Observational Studies (QATSO) Score:

Five item for quality indicator

1) External validity (1 item) – addresses the extent to which the findings from the study can be generalised to the population from which the study subjects are derived.

2) Reporting (2 items) – assesses whether the information provided in the paper is sufficient to allow a reader to make an unbiased assessment of the findings of the study. One of the items is specific for prevalence studies.

3) Bias (1 item) – addresses bias in the measurement of the outcomes in a study.

4) Confounding (1 item) – addresses whether studies have applied adjustment for confounding in the analysis. This item is specific to studies concerning association of risk factors.

Although the QATSO Score consists of five items, users may select 4–5 items depending on the type of studies being evaluated. Studies achieving 67% or more in the score will be regarded as "good" quality; 34–66% "fair"; and, below 33% "poor".
